# Postoperative pulmonary complications and outcomes in cytoreductive surgery for ovarian cancer: a propensity-matched analysis

**DOI:** 10.1186/s12871-022-01660-2

**Published:** 2022-04-23

**Authors:** Mengmeng Xu, Wei Zhang, Chen Gao, Ying Zhou, Yanhu Xie

**Affiliations:** 1 Department of Anesthesiology, The First Affiliated Hospital of USTC, Division of Life Sciences and Medicine, University of Science and Technology of China, Hefei, 230001 Anhui China; 2 Department of Obstetrics and Gynecology, The First Affiliated Hospital of USTC, Division of Life Sciences and Medicine, University of Science and Technology of China, Hefei, 230001 Anhui China

**Keywords:** Cytoreductive surgery, Postoperative pulmonary complications, Outcome, Diaphragmatic resection

## Abstract

**Objectives:**

To assess the prevalence of postoperative pulmonary complications (PPCs), the perioperative factors associated with PPCs, and the association of PPCs with postoperative outcomes in ovarian cancer patients undergoing cytoreductive surgery.

**Methods:**

A retrospective analysis was conducted on patients who underwent cytoreductive surgery in our hospital, between September 2017 and January 2021, and patient medical records were reviewed to collect relevant clinical information. Univariable and multivariable analyses were used to identify significant risk factors for PPCs. Analysis of the association of PPCs with postoperative outcomes, mortality and 30-day readmission, was undertaken utilizing propensity score-matched controls and multivariable logistic regression model.

**Results:**

Final analysis was performed with 268 ovarian cancer patients after cytoreductive surgery, among whom the incidence of PPCs was 26.9%, and the most frequent pulmonary complication was pleural effusion. According to the multivariate analysis, the intraoperative fluid infusion volume (L) (odds ratio (OR) 1.34; 95% confidence intervals (CI) 1.01–1.77; *P* = 0.040), diameter size of diaphragmatic resection (cm) (OR 1.16; 95% CI 1.06–1.28; *P* = 0.002), and surgical complexity scores (OR 1.26; 95% CI 1.13–1.42; *P* < 0.001) were significantly associated with the development of PPCs. The multivariable logistic regression analyses with propensity-matched controls demonstrated that the occurrence of PPCs significantly increased the risk of 30-day readmission (OR 6.01; 95% CI 1.12–32.40; *P* = 0.037) and did not significantly affect inpatient mortality.

**Conclusion:**

Ovarian cancer patients undergoing cytoreductive surgery, especially those with diaphragmatic resection or higher surgical complexity scores, represent a high-risk population for PPCs. In addition, goal-directed fluid therapy is vital to reducing the occurrence of PPCs in patients at risk. PPCs were not associated with in-hospital mortality but were significantly associated with an increased risk of 30-day readmission after cytoreductive surgery.

**Supplementary Information:**

The online version contains supplementary material available at 10.1186/s12871-022-01660-2.

## Introduction

Ovarian cancer is the most prevalent malignant tumor in gynecology [1]. Cytoreductive surgery is the main effective method of clinical treatment for advanced ovarian cancer. Optimal debulking surgery can improve the prognosis and survival of patients with ovarian cancer [2, 3]. Although perioperative management and surgical techniques are developing rapidly, postoperative complications may still be inevitable. A systematic review reported that the major complication rate was 23% for advanced ovarian cancer surgery [4]. Several findings have indicated that pleural effusion is the most common complication after cytoreduction for advanced stage epithelial ovarian cancer [5, 6]. Postoperative pulmonary complications (PPCs), are the most prevalent complication affecting the respiratory system after anesthesia and surgery. Even mild PPCs can increase early postoperative mortality, the intensive care unit (ICU) admission rate and lengthen the duration of hospitalization [7]. However, there have not yet been any studies investigating the relationship between PPCs and outcomes after cytoreductive surgery.

The risk factors for PPCs development are complicated [8], and clinicians should pay attention to alterable and unalterable factors to identify high-risk patients and optimize their care. In general, factors for PPCs can be divided into patient-related (age, co-morbidity, smoking, laboratory testing), intraoperative (surgery type, anesthesia means, mechanical ventilation strategy, intraoperative blood transfusion), and postoperative factors (postoperative analgesia and mobilization). The current literature is primarily limited to the analysis of surgical-related factors of cytoreductive surgery. Therefore, this present study included perioperative relevant indicators to explore the incidence and risk factors associated with PPCs after cytoreductive surgery. Then, we evaluated the association of PPCs as an exposure variable with short-term postoperative outcomes, in-hospital mortality and readmission.

## Materials and methods

### Patients

This was a monocentric retrospective study. Data from patients who underwent cytoreductive surgery at the First Affiliated Hospital of the University of Science & Technology of China from September 2017 to January 2021 were retrospectively collected. We enrolled patients who received satisfactory cytoreduction surgery and were pathologically diagnosed as having ovarian cancer after surgery. Patients without macroscopic residual lesions or with residual lesions less than 1 cm were defined as having underwent satisfactory cytoreductive surgery. Patients were excluded for preoperative pulmonary complications such as pleural effusion, pulmonary infection or pulmonary embolism.

### Data collection

Data included patient characteristics, American Society of Anesthesiologists (ASA) grade, previous comorbidities, pulmonary function, preoperative and postoperative albumin (Alb),  hemoglobin (Hb), intraoperative fluid infusion volume, blood transfusion volume, blood loss volume, anesthesia means, operation time, surgical methods for diaphragmatic lesions, Federation International of Gynecology and Obstetrics (FIGO) stage, surgical complexity scores (SCS) [9], and hospitalization days.

In cytoreductive surgery for ovarian cancer, the commonly used surgical methods for the diaphragmatic lesions mainly include the following: electrocoagulation and cauterization of simple diaphragmatic lesions, diaphragm peritonectomy (DP; stripping) (removal of diaphragmatic peritoneum only) and diaphragm full-thickness resection (DFTR) (resection of diaphragmatic peritoneum and muscle layer) in case of infiltration.

The primary outcome was defined as the incidence of PPCs within 30 days after surgery. Pulmonary complications included pleural effusion, pneumothorax, atelectasis, pneumonia, pulmonary embolism, postoperative mechanical ventilation > 48 h, acute respiratory distress syndrome, re-intubation or respiratory failure. The diagnostic criteria of PPCs used in this study were based on the European Perioperative Clinical Outcome (EPCO) definitions for postoperative pulmonary complications [10].

Secondary outcomes included in-hospital mortality and 30-day readmission. In-hospital mortality was defined as death during admission at our hospital. 30-Day readmission also only included admission back to our hospital. Planned admissions for administration of chemotherapy or reexamination were not considered to be a 30-day readmission event.

### Statistical analysis

Categorical variables were presented as number with percentage, and continuous variables were either presented as mean (SD) or median (IQR). Chi-square (χ^2^) tests or Fisher’s exact tests were used for categorical variables, and continuous variables were analyzed using Student t-test or Mann-Whitney U test where appropriate. The associations between different variables were evaluated using univariable and multivariable logistic regression analyses, and the odds ratio (OR) with 95% confidence interval (CI) was calculated. A receiver operating characteristic (ROC) analysis was performed to evaluate the area under the curve (AUC) with 95% CI of risk factors for PPCs.

Due to the observed imbalance in the sample size between the 2 groups, we fit a propensity score model to assess the association of PPCs with inpatient mortality and readmission. The propensity score calculated for each observation object is a measure of the probability that a patient would have experienced PPCs. The propensity score was derived from a logistic regression with PPCs as outcome using all terms in Table 1. Patients with similar propensity scores (with caliper of 0.1) were matched in a 1:3 ratio to compare outcomes among patients who did develop PPCs to patients who did not develop PPCs. These groups were determined to be well matched with a standardized difference of < 10%. We then ran multivariable logistic regression models using our propensity-matched data with PPCs as a covariate. For all statistical analyses, IBM SPSS version 22.0 (IBM Corp., Armonk, New York, USA) was used, and a *P* value less than 0.05 was considered statistically significant.

**Table 1:** Clinical characteristics of ovarian cancer patients

Clinical Characteristics	PPCs Group	Non-PPCs Group	*P* value
Number	72 (26.9%)	196 (73.1%)	
Age (Y)^a^	55.0 ± 8.6	55.1 ± 10.7	0.035
BMI (kg/m^2^)^a^	23.0 ± 3.2	23.1 ± 3.1	0.954
ASA grade			0.268
II	1 (1.4%)	4 (2.0%)	
III	58 (80.6%)	171 (87.2%)	
IV	13 (18.1%)	21 (10.7%)	
Preoperative comorbidities			
Hypertension	16 (22.2%)	31 (15.8%)	0.222
Diabetes	6 (8.3%)	17 (8.7%)	0.930
Heart disease	0 (0.0%)	7 (3.6%)	0.195
Preoperative DVT	4 (5.6%)	10 (5.1%)	0.547
COPD	2(2.8%)	2 (1.0%)	0.293
Obsolete pulmonary tuberculosis	0 (0.0%)	1 (0.5%)	0.731
Smoking history	1 (1.4%)	1 (0.5%)	0.466
Pulmonary function (L)^b^			
FEV_1_	2.27 (2.15–2.37)	2.25 (2.15–2.35)	0.415
Neo-adjuvant chemotherapy	4 (5.6%)	21 (10.7%)	0.206
Preoperative hemoglobin (g/L)^a^	116.9 ± 12.9	115.7 ± 16.1	0.041
Preoperative albumin (g/L)^a^	39.8 ± 4.6	41.6 ± 4.7	0.693
Intraoperative fluid infusion volume (L)^b^	3.5 (3.0–4.0)	2.5 (2.0–3.5)	< 0.001
Blood transfusion volume (L)^b^	0.8 (0.6–1.6)	0.6 (0.0–0.8)	< 0.001
Blood loss volume (L)^b^	1.0 (0.8–1.6)	0.8 (0.4–1.0)	< 0.001
Operation time (h)^a^	5.8 ± 1.8	4.2 ± 1.5	0.164
Surgery methods			0.059
Primary cytoreductive surgery	61 (84.7%)	139 (70.9%)	
Intermediate cytoreductive surgery	3 (4.2%)	22 (11.2%)	
Re-cytoreductive surgery	8 (11.1%)	35 (17.9%)	
Diaphragmatic surgery			< 0.001
No diaphragmatic lesions	12 (16.7%)	122 (62.2%)	
Electrocoagulation/cauterization	5 (6.9%)	19 (9.7%)	
DP/stripping	19 (26.4%)	37 (18.9%)	
DFTR with direct closure	12 (16.7%)	10 (5.1%)	
DFTR with patch	24 (33.3%)	8 (4.1%)	
Maximum diameter size of diaphragmatic resection (cm)^b^	0.5 (0.0–8.0)	0.0 (0.0–0.0)	< 0.001
SCS^b^	9 (6–13)	5 (4–7)	< 0.001
Residual disease			< 0.001
0	60 (83.3%)	174 (88.8%)	
< 1 cm	12 (16.7%)	22 (11.2%)	
FIGO stage			< 0.001
I	1 (1.4%)	16 (8.2%)	
II	1 (1.4%)	40 (20.4%)	
III	53 (73.6%)	122 (62.2%)	
IV	17 (23.6%)	18 (9.2%)	
Anesthesia means			0.302
GA	13 (18.1%)	47 (24%)	
GA combined with TAP block	59 (81.9%)	149 (76%)	
Postoperative albumin (g/L)^a^	29.0 ± 5.0	32.0 ± 4.8	0.632
Admission to ICU after operation	19 (26.4%)	21 (10.7%)	0.001
Hospitalization characteristics			
ICU length of stay (d)^b^	0.0 (0.0–1.0)	0.0 (0.0–0.0)	0.002
Length of stay (d)^b^	26.0 (19.0–34.5)	17.5 (14.0–23.0)	< 0.001
Outcomes			
Mortality, in-hospital	2 (2.8%)	0 (0.0%)	0.071
Readmission, 30d	8 (11.1%)	5 (2.6%)	0.008

*PPCs* Postoperative pulmonary complications, *BMI* Body mass index, *ASA* American Society of Anesthesiologists, *DVT* Deep vein thrombosis, *COPD* Chronic obstructive pulmonary disease, *FEV*_*1*_ forced expiratory volume in 1 s, *DP* diaphragm peritonectomy, *DFTR* diaphragm full-thickness resection, *SCS* Surgical complexity scores, *FIGO* Federation International of Gynecology and Obstetrics, *GA* general anesthesia, *TAP block* transversus abdominis plane block

^a^Data are presented as mean ± standard deviation (SD)

^b^Data are presented as median, interquartile range (IQR)

## Results

### Patient characteristics

A total of 268 patients who received optimal debulking surgery for ovarian cancer were analyzed in the present study. The mean age was 55.1 ± 10.2 years, and the majority of patients (74.6%) were treated with primary cytoreductive surgery. Other baseline characteristics are shown in Table 1. In this study, 134 patients (50%) had metastatic lesions of the diaphragm and underwent diaphragmatic surgery, of whom 56 patients (41.8%) underwent peritonectomy of the diaphragm, 22 patients (16.4%) underwent full thickness resection that was directly closed and 32 patients (23.9%) underwent full thickness resection that needed closure with a patch. Diaphragmatic resection was present in 20.1% of the OC patients, and the median maximum diameter size was 8 cm (IQR 5–10 cm). The median SCS for debulking surgery was 6 (IQR 4–8.75).

PPCs occurred in 72 OC patients (26.9%) within 30 days after surgery, the most frequent pulmonary complication was pleural effusion (22.8%), followed by atelectasis (8.2%), pulmonary embolism (6.3%) and others (Table 2). Two patients (0.7%) died in the hospital after surgery. In detail, one patient died of multi organ failure from sepsis caused by intestinal fistula, and one patient died because of disseminated intravascular coagulation (DIC) caused by hemorrhagic shock.

**Table 2:** Incidence of PPCs after cytoreduction surgery for ovarian cancer patients

PPCs	N (%)
Pleural effusion	60 (22.4%)
Pneumothorax	5 (1.9%)
Pneumonia	17 (6.3%)
Atelectasis	22 (8.2%)
Pulmonary embolism	17 (6.3%)
Postoperative mechanical ventilation > 48 h	7 (2.6%)
Unplanned re-intubation	3 (1.1%)

Data are presented as number of patient (%)

*PPCs* Postoperative pulmonary complications

### Perioperative factors associated with PPCs

**Fig. 1:**
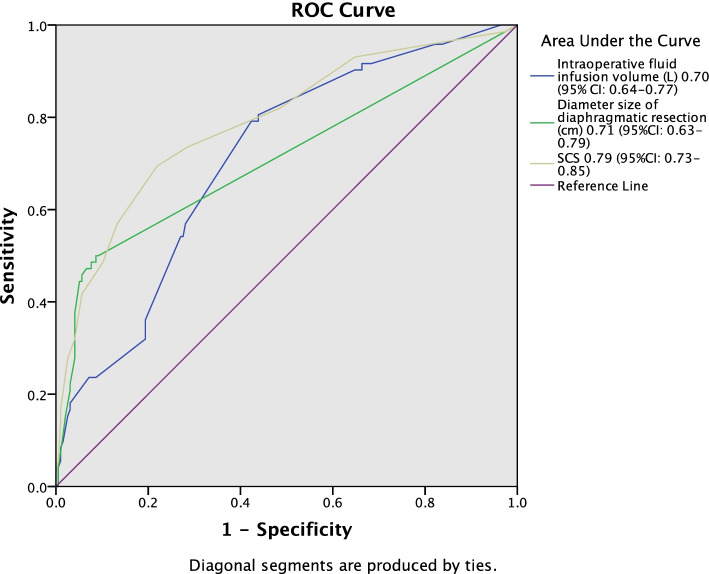
ROC curve of predict risk factors of PPCs after cytoreduction surgery for ovarian cancer patients

The results from univariable and multivariable risk factor analysis are listed in Table 3. We found that pre- and postoperative albumin, intraoperative fluid infusion volume (L), blood transfusion volume (L), blood loss volume (L), operation time, diaphragmatic surgery, diameter size of diaphragmatic resection, SCS and FIGO stage were associated with PPCs according to the univariable analysis. Then the multivariable analysis model was used to identify the independent risk factors of PPCs. Intraoperative fluid infusion volume (L), diameter size of diaphragmatic resection and SCS were maintained in the model after adjustment. ROC curve analysis was performed to assess the diagnostic value of different risk factors for PPCs. SCS is the most valuable predictor, the area under the ROC curve (AUC) was 0.79 (95% CI 0.73–0.85), the sensitivity was 69.4%, and the specificity was 78.1% (Fig. 1).

**Table 3:** Univariable and multivariable analysis of factors associated with PPCs after cytoreduction surgery for ovarian cancer patients

	Univariate analysis		Multivariate analysis	
	OR (95%CI)	*P* value	OR (95%CI)	*P* value
Age (Y)	0.99 (0.97–1.03)	0.948		
BMI (kg m^−2^)	0.99 (0.91–1.08)	0.849		
ASA grade	1.77 (0.87–3.59)	0.116		
Preoperative comorbidities				
Hypertension	1.52 (0.77–2.99)	0.224		
Diabetes	0.96 (0.36–2.53)	0.930		
Preoperative DVT	1.09 (0.33–3.61)	0.882		
Preoperative albumin (g L^−1^)	0.92 (0.87–0.98)	**0.006**		
Preoperative hemoglobin (g L^−1^)	1.01 (0.99–1.02)	0.588		
Pulmonary function				
FEV_1_ (L)	1.97 (0.51–7.63)	0.328		
Neo-adjuvant chemotherapy	0.49 (0.16–1.48)	0.206		
Intraoperative fluid infusion volume (L)	1.79 (1.39–2.29)	**< 0.001**	1.34 (1.01–1.77)	**0.040**
Blood transfusion volume (L)	1.89 (1.32–2.70)	**0.001**		
Blood loss volume (L)	1.47 (1.14–1.89)	**0.003**		
Operation time (h)	1.81 (1.48–2.20)	**< 0.001**		
Diaphragmatic surgery		**< 0.001**		
Electrocoagulation/cauterization	2.68 (0.85–8.45)	0.093		
DP/stripping	5.22 (2.32–11.75)	**< 0.001**		
DFTR with direct closure	12.20 (4.37–34.09)	**< 0.001**		
DFTR with patch	30.50 (11.27–82.57)	**< 0.001**		
Diameter size of diaphragmatic resection (cm)	1.31 (1.19–1.44)	**< 0.001**	1.16 (1.06–1.28)	**0.002**
SCS	1.42 (1.29–1.57)	**< 0.001**	1.26 (1.13–1.42)	**< 0.001**
FIGO stage	3.29 (1.96–5.51)	**< 0.001**		
Anesthesia means	1.43 (0.72–2.84)	0.304		
Postoperative albumin (g L^− 1^)	0.89 (0.84–0.94)	**< 0.001**		

*PPCs* Postoperative pulmonary complications, *OR* Odds ratio, *CI* Confidence intervals, *BMI* Body mass index, *ASA* American Society of Anesthesiologists, *DVT* Deep vein thrombosis, *COPD* Chronic obstructive pulmonary disease, *FEV*_*1*_ Forced expiratory volume in 1 s, *DP* diaphragm peritonectomy, *DFTR* diaphragm full-thickness resection, *SCS* Surgical complexity scores, *FIGO* Federation International of Gynecology and Obstetrics, *GA* general anesthesia, *TAP block* transversus abdominis plane block

### Association of PPCs with short-term outcomes

To examine the association between PPCs and postoperative short-term outcomes, propensity score matching was used to create matched PPCs and non-PPCs cohorts (Supplemental Table 1). A 1-to-3 match was achieved for the 42 patients who developed PPCs and 91 matched patients who did not develop PPCs. After propensity-matched adjustment, logistic regression analysis demonstrated that there was no significant effect on mortality for patients who developed PPCs compared with patients who did not (*P* = 0.098). And patients who developed PPCs had an increased risk of 30-day readmission, the estimated OR of 30-day readmission was 6.01 times higher for patients who did not (OR 6.01; 95% CI 1.12–32.40; *P* = 0.037) (Table 4).

**Table 4:** Propensity-Matched Adjusted Outcomes

Propensity-Matched Cohorts	PPCs *n* = 42	Non-PPCs *n* = 91	OR (95% CI)	*P* value
Mortality, in-hospital	2 (4.8%)	0 (0.0%)	6.92 (0.70–68.65)	0.098
Readmission, 30d	5 (11.9%)	2 (2.2%)	6.01 (1.12–32.40)	**0.037**

*PPCs* Postoperative pulmonary complications, *OR* Odds ratio, *CI* Confidence intervals

## Discussion

Previous research indicated that the quality of ovarian tumor cytoreductive surgery is an independent predictor of patient prognosis [11, 12]. Bristow et al. [13] reported that a 10% increase in the optimal cytoreduction rate prolongs the median survival time by 5.5%. To accomplish optimal debulking surgery, extensive abdominal surgery is inevitable. Extensive upper abdominal surgery can decrease the residual disease rate in ovarian cancer patients, and may also increase the incidence of postoperative complications [14–16]. In different medical centers, the proportion of patients with advanced ovarian cancer receiving optimal debulking surgery varies widely in previous studies, from 15 to 85% [13]. Therefore, based on the level of ovarian cancer cytoreductive surgery and perioperative management in our center, we analyzed PPC events to optimize the perioperative management of patients in our hospital. The incidence of PPCs in this study was 26.9%. Some authors have seen relatively high rates of early pulmonary complications (32.3%) after cytoreductive surgery [5]. Pleural effusion was the most common PPC in this cohort, a total of 60 cases, of which nearly half (48.3%) need pleural puncture drainage. Previous studies have shown that the need for pleural puncture or secondary drainage prolongs hospital stay and postoperative pain [17]. We found that the occurrence of PPCs prolonged the length of stay but did not increase mortality. Moreover, the propensity-matched analysis found an estimated 6 times higher risk of 30-day readmission among patients who developed PPCs after cytoreductive surgery. These results indicate that PPC is a highly common, and possibly underappreciated, complication in patients undergoing debulking surgery.

In advanced ovarian cancer patients with FIGO stage IIIC/IV, diaphragmatic involvement is a common metastatic site. Intraoperative diaphragmatic evaluation has been suggested for all patients undergoing cytoreductive surgery for advanced ovarian cancer [18, 19]. Patients who underwent diaphragmatic surgery were more likely to develop pleural effusion within 3 days after surgery [6, 20]. In our center, patients who received DFTR with patch routinely placed a thoracic drainage tube intraoperatively to prevent postoperative pleural effusion. There is not enough evidence to justify prophylactic chest tube placement for all patients [6]. It is recommended to routinely perform chest radiography or CT 3 days after surgery to evaluate pleural effusion or other pulmonary complications. According to the result of previous studies, risk factors for the occurrence of PPCs were liver mobilization [21, 22], pleural opening [23], the size of the diaphragmatic resection [17, 22]. Based on our research, in addition to the size of diaphragmatic resection, intraoperative fluid infusion volume and SCS are also independent risk factors for pulmonary complications after cytoreductive surgery. Some researchers have also noticed that OC patients receiving DFTR are more likely to develop PPCs than patients receiving DP [6]. Diaphragmatic surgery was significantly associated with PPCs in univariable models. After adjustment, only the size of diaphragmatic resection was found to be significantly associated with PPCs. This could be mainly due to the strong collinearity between these two variables.

As a matter of fact, diaphragmatic resection and SCS are reflections of tumor extent indirectly. SCS was the factor most predictive of PPCs in our work. Prior research on neoadjuvant chemotherapy has demonstrated that preoperative chemotherapy can reduce the extent of surgery and complications in patients with ovarian cancer [24, 25]. Such differences were not seen in our study on pulmonary complications. Cytoreductive surgery results in functional disruption of respiratory muscles, including the diaphragm, airway muscles and abdominal muscles [26], which leads to the decline of postoperative pulmonary function. In addition, diaphragm defect, abdominal exudation and postoperative inflammatory mediators release were suggested to be possible mechanisms of pulmonary complications [21, 27].

Given the high morbidity rate after diaphragmatic surgery, we need to explore the clinical indicators that can be controlled to minimize the incidence rate of PPCs. Our results show that intraoperative fluid infusion volume (L) was significantly associated with PPCs, and patients undergoing debulking surgery are at an increased risk of PPCs with increased fluid infusion. Excessive infusion during operation can reduce plasma colloid osmotic pressure, further facilitating the aggregation of pulmonary edema and the decline of oxygenation capacity. Generally, massive fluid resuscitation may be a reflection of more intraoperative bleeding during the procedure, or a reflection of high surgical complexity that consumes considerable surgical time. There are studies have shown that goal-directed fluid therapy (GDFT) can reduce postoperative complications after abdominal surgery [28, 29]. Although our anesthesiology group, there has been an emphasis on goal-directed fluid management. Whereas, perioperative GDFT this consensus embraces a variety of strategies [30]. In any case, the study of an optimum therapy strategy in OC patients is an area with much work still to be done.

Postoperative severe pain may lead to shallow breathing and even atelectasis, regional anesthesia would be better than intravenous opioids for pain management, can result in improved postoperative pulmonary function [31]. Transversus abdominis plane (TAP) blocks, place local anesthetic into the neurovascular plane between the internal oblique and transversus abdominis muscle blocking the sensory nerves of the anterior rami of the lower thoracic nerve (T7-T12) and the first lumbar nerve (L1), and providing effective postoperative analgesia [32]. In this study, most patients were routinely treated with TAP block for postoperative analgesia. Unfortunately, no significant intergroup difference was found between GA combined with TAP block or not. We believe that these results may be important for future research to better improve the prognosis of OC patients.

As a retrospective study, there are several limitations. Firstly, ovarian cancer patients included in this study were confined to a single medical center. As the medical conditions and environmental quality of hospitals differ, this limits the scalability of the results. Second, the information of mechanical ventilation parameters was not taken into account because the electronic management system could not record them in time. Finally, all patients in our center received patient-controlled analgesia (PCA) pump after cytoreductive surgery, and sufentanil was the key formulation for PCA. We also applied multimodal analgesic regime to reduce opioid consumption. To our knowledge, there is no report focusing on opioid-free analgesia in postoperative pain management after cytoreduction. New studies and data are required to elaborate the optimal analgesic approach for cytoreductive surgery.

Ovarian cancer patients undergoing cytoreductive surgery, especially those with diaphragmatic resection or higher surgical complexity scores, represent a high-risk population for PPCs. In addition, goal-directed fluid therapy is vital to reducing the occurrence of PPCs in patients at risk. Although PPCs were unrelated to in-hospital mortality, the occurrence of PPCs was significantly related to an increased risk of 30-day readmission after cytoreductive surgery. More effective perioperative management strategy is necessary for ovarian cancer patients.

## Supplementary Information

**Additional file 1: Supplement Table 1.** Propensity-matching

## Data Availability

The data that support the findings of this study are available on request from the corresponding author.
