# Supplementary material for: Postoperative pulmonary complications and outcomes in cytoreductive surgery for ovarian cancer: a propensity-matched analysis
Source: BMC Anesthesiol. 2022 Apr 23;22:120. doi: 10.1186/s12871-022-01660-2 (PMC9034613; doi:10.1186/s12871-022-01660-2)
Supplement: Supplementary file 1 — Additional file 1: Supplement Table 1. Propensity-matching [file 12871_2022_1660_MOESM1_ESM.docx]

**Supplement Table 1** Propensity-matching

| Clinical Characteristics | PPCs  n = 42 | Non-PPCs  n = 91 | Standard mean difference |
| --- | --- | --- | --- |
| Age (Y) | 55.52 (7.33) | 55.47 (9.74) | 0.002 |
| BMI (kg/m^2^) | 23.24 (3.32) | 23.25 (3.04) | 0.031 |
| ASA grade | 3.14 (0.42) | 3.11 (0.38) | 0.050 |
| Neo-adjuvant chemotherapy | 0.05 (0.22) | 0.05 (0.23) | 0.034 |
| Preoperative albumin (g/L) | 39.76 (4.09) | 40.18 (5.35) | 0.033 |
| Intraoperative fluid infusion volume (L) | 3.37 (1.19) | 3.26 (1.22) | 0.007 |
| Blood transfusion volume (L) | 0.98 (0.96) | 0.94 (0.89) | 0.020 |
| Blood loss volume (L) | 1.25 (1.23) | 1.24 (1.32) | 0.005 |
| Operation time (h) | 4.98 (1.31) | 4.84 (1.59) | 0.027 |
| Diameter size of diaphragmatic resection (cm) | 2.01 (3.78) | 1.29 (3.28) | 0.028 |
| SCS | 7.83 (3.02) | 6.95 (2.85) | 0.034 |
| Residual disease | 0.19 (0.40) | 0.13 (0.34) | 0.071 |
| FIGO stage | 3.12 (0.55) | 3.07 (0.47) | 0.067 |

Data reported as mean (standard deviation).

PPCs, Postoperative pulmonary complications; BMI, Body mass index; ASA, American Society of Anesthesiologists; SCS, Surgical complexity scores; FIGO, Federation International of Gynecology and Obstetrics.
